# Physical frailty prediction model for the oldest old^1^

**DOI:** 10.1590/1518-8345.2346.3023

**Published:** 2018-09-06

**Authors:** Jacy Aurelia Vieira de Sousa, Maria Helena Lenardt, Clóris Regina Blanski Grden, Luciana Kusomota, Mara Solange Gomes Dellaroza, Susanne Elero Betiolli

**Affiliations:** 2 PhD, Adjunct Professor, Departamento de Enfermagem e Saúde Pública, Universidade Estadual de Ponta Grossa, Ponta Grossa, PR, Brazil.; 3 PhD, Professor, Departamento de Enfermagem, Universidade Federal do Paraná, Curitiba, PR, Brazil.; 4 PhD, Professor, Escola de Enfermagem de Ribeirão Preto, Universidade de São Paulo, PAHO/WHO Collaborating Centre for Nursing Research Development, Ribeirão Preto, SP, Brazil.; 5 PhD, Adjunct Professor, Departamento de Enfermagem, Universidade Estadual de Londrina, Londrina, PR, Brazil.; 6 PhD, Professor, Departamento de Enfermagem, Sociedade Educacional Herrero, Curitiba, PR, Brazil.

**Keywords:** Aged, Aged, 80 and Over, Frail Elderly, Aging, Geriatric Nursing, Morbidity

## Abstract

**Objective:**

to present a physical frailty prediction model for oldest old users of primary health care, according to clinical variables.

**Method:**

cross-sectional study with proportional stratified sample of 243 oldest old subjects. Data were collected through a structured clinical questionnaire, handgrip strength test, walking speed, weight loss, fatigue/exhaustion, and physical activity level. For the analysis of the data, univariate and multivariate analysis by logistic regression were used (p<0.05), which resulted in prediction models. The odds ratios (95% Confidence Interval) of the models were calculated. Each model was evaluated by deviance analysis, likelihood ratios, specificity and sensitivity, considering the most adequate. All ethical and legal precepts were followed.

**Results:**

the prediction model elected was composed of metabolic diseases, dyslipidemias and hospitalization in the last 12 months.

**Conclusion:**

clinical variables interfere in the development of the physical frailty syndrome in oldest old users of basic health unit. The choice of a physical frailty regression model is the first step in the elaboration of clinical methods to evaluate the oldest old in primary care.

## Introduction

Senescence is characterized by inevitable structural, physiological, and functional changes in the organism. For some people, these changes are accentuated and lead to increased risk of morbidity and mortality, while others remain robust, even in old age. Given the heterogeneity of the aging process, the concept of frailty has been increasingly discussed.

Physical frailty is a multicausal medical condition with several associated factors. It is characterized by a decrease in strength and endurance and an increase in the individual’s vulnerability for developing increased dependency and/or mortality^1^. This syndrome is an important marker of an individual’s physiological reserve and an indicator of the risk of negative outcomes to the health of the oldest-old^2^ ^-^ ^3^. 

Aiming to construct a phenotype of frailty, international authors developed a model based on the markers decrease in handgrip strength, self-reported exhaustion or fatigue, diminished walking speed, unintentional weight loss and low level of physical activity^4^. Older adults without any of the markers are considered non-frail, those with one or two markers are called pre-frail and the presence of three or more markers characterizes frail older adults.

The oldest-old are characterized as a group that should be screened, even without evidence of disability^1^ ^,^ ^5^ ^-^ ^6^. The high prevalence of physical frailty and the increase in the demand for health services among the oldest-old has stimulated discussions for the definition of predictors to better evaluate, characterize and monitor this age group^7^. 

Among the factors related to the development and worsening of the frailty syndrome, the most prominent are clinical factors. An international cross-sectional study with 115 participants aged 65 and over in the Singapore University Hospital highlighted the association between the syndrome and recurrent hospital admissions, polypharmacy, and falls^8^. Another international longitudinal study conducted with 2,925 Italian older adults with a mean age of 74.4 years showed that clinical variables, such as polypharmacy, chronic diseases and obesity, may worsen the frailty state^9^. Similar results were obtained in a national cross-sectional study carried out with 385 independent older adults in the city of Ribeirão Preto, São Paulo, which found that frail older adults had a greater chance of having had a hospitalization in the prior 12 months, had more medical visits, and had more cases of cerebrovascular events, diabetes, urinary and fecal incontinence, osteoporosis and neoplasms^10^.

The identification of clinical factors associated with adverse outcomes for the health of older adults and the careful evaluation of the markers of physical frailty are essential for an adequate management of the syndrome, with the elaboration of effective interventions in the care of older adults. 

One of the possible strategies for screening for physical frailty among older adults is the use of prediction models. International authors point out that this is a simple and clinically relevant tool that allows the use of routinely collected data in a systematic manner, optimizing data quality and reliability^11^. For nurses in primary care, strategies like this can increase the speed and effectiveness of the care provided to the older adult.

The present study aimed to present a physical frailty prediction model for oldest-old patients of primary health care according to clinical variables.

## Method

Cross-sectional study conducted in households in the area covered by three Basic Health Units (BHU) of the city of Curitiba, Paraná. The criteria for choosing the BHU were: having users belonging to the income classes C, D and E^12^, since the classes A and B are not included in the BHU care; and having a significant number of older adults registered. The study population consisted of older adults aged 80 years or over and registered in these BHU. 

Proportional stratified sampling was adopted considering that none of the BHU was overestimated or underestimated. The sample calculation considered a beta power of 80% (1-ß), a 5% significance level(α=0.05) and a minimum significant difference of 10% between the proportions of elderly individuals with the syndrome. From the total of 503 older adults, 10% were added to the sample size due to the possibilities of losses and refusals, which resulted in a final sample of 243 older adults.

The selection of the participants was random, through draw from the list of oldest-old patients enrolled in the selected BHU. For each participant, a maximum of three attempts to visit were made. In case of refusal, impossibility of participation or absence from the household, another participant was drawn, until reaching the sample determined for each BHU.

The following inclusion criteria were established for the participants: (a)being 80 years old or older; (b)being registered in one of the BHU of the research; (c)scoring higher than the cut-off in the cognitive test of the Mini-Mental State Examination (MMSE)^13^ considering 13 points as illiterate, 18 as low (1 to 4 incomplete years) and average (4 to 8 incomplete years) education level and 26 as high education level (8 years or more)^14^. Older adults undergoing chemotherapy or with previous diagnosis of serious mental illness or deficits that prevented participation in the study were excluded.

In the case of older adults with no cognitive conditions to answer the research questions (n​​=36) at this stage, the family caregiver was invited to participate, for which the following inclusion criteria were adopted: a) being 18 years or older; b) being a family caregiver; c) be living with the older adult for at least three months.

Data were collected from January 2013 to September 2015, in the household of the participants, through a structured clinical questionnaire, application of scales and physical tests that make up the evaluation of physical frailty. The data collection was carried out by scientific initiation undergraduate students and master and doctoral students, after previous training. A pilot study with ten oldest-old individuals was carried out to verify and adapt the questionnaire.

The clinical questionnaire consisted of specific questions about the clinical aspects of the oldest-old, inspired by sections II (Physical health) and III (Use of medical and dental services) of the multidimensional questionnaire Brazil Old Age Schedule (BOAS), elaborated and validated for evaluation of the older adult population of a large Brazilian urban center^15^. The following clinical variables were investigated: diseases, falls in the last 12 months, hospitalizations in the last 12 months and use of medications. 

The markers of the syndrome were evaluated based on the phenotype of frailty^4^, with some adaptations. 

**Figure 1:**
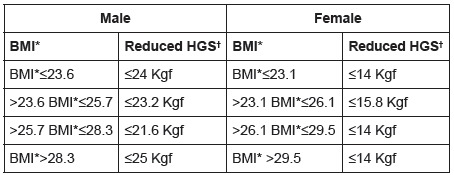
Cut-off points for handgrip strength adjusted for gender and body mass index of the participants. Curitiba, PR, Brazil, 2015

Handgrip strength (HGS) was measured using a Jamar® hydraulic dynamometer. Three measurements in kilogram/force (Kgf) were taken with the dominant hand, with one-minute intervals to regain strength and the highest reading was recorded^16^. Values ​​were adjusted according to gender and body mass index (BMI, in Kg/m^2^), considering the values ​​in the lowest quintile as markers of physical frailty (Figure 1).

* BMI - Body Mass Index; † HGS - Handgrip strength

To evaluate walking speed (in m/s), the participants were instructed to walk a distance of six meters in their usual pace on a flat surface, signaled by two marks distant four meters from each other. In order to reduce acceleration and deceleration effects, the first and last meters were not timed, only the four-meter course was considered. An international literature review study evaluating walking speed tests, pointed out that six-meter courses have been widely used with older adults and that 4 to 6-meter courses can be used, according to the purpose of the study^17^.

**Figure 2:**
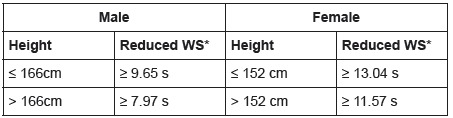
Cut-off points for walking speed adjusted according to gender and height of the participants. Curitiba, PR, Brazil, 2015

After adjusting for gender and height, values equal or higher than the cutoff points were considered frailty markers (Figure 2).

* WS - Walking Speed

Weight loss was verified through the self-report of the participant on the following questions: a) Did you lose weight in the last twelve months? b) If yes, how many kilograms? Unintentional weight loss equal to or greater than 4.5 kg in the prior twelve months was considered as a marker for physical frailty.

The marker fatigue/exhaustion was verified based on the self-report of the participant on the question “Do you feel full of energy?”, present on the Depression Scale of the Center for Epidemiological Studies^18^. A negative response to the question represented a marker of frailty.

The Physical Activity Level Questionnaire for the Elderly - CuritibAtiva was used to evaluate the level of physical activity of the participants. This questionnaire contains twenty questions related to the frequency and time of activities performed in the last week by the older adult and at the end of the evaluation it classifies the subject as inactive (0-32 points), not very active (33-82 points), moderately active (83-108 points), active (109-133 points) or very active (134 points or more)^19^. The classifications of inactive or not very active, according to the instrument, were considered frailty markers.

Statistical analyzes were performed in the software Statistica10. For the clinical characterization of the sample, descriptive analyzes were performed using absolute and percentage frequency distribution, mean and standard deviation, as well as other measures of central tendency (mode and median).

The univariate analysis was performed using the chi-square test, with p value<0.05. Each clinical variable was evaluated separately in relation to the response of interest - the frailty. In the multivariate analysis through logistic regression, two groups were analyzed (Cluster analysis), with joint analysis of the categories Pre-frail and Non-Frail. The Pre-frail and Non-Frail categories were analyzed together because the logistic regression is basically limited to two groups. The classification of frail was determined as priority response (event of interest) and the other category, Non-Frail, was considered its complement, according to a model associated with binomial distribution.

For the elaboration of the prediction model, all clinical variables of the study were initially included; then, the forward stepwise method was used to include those individual data that presented lower p-value. The respective odds ratio (OR) and 95% confidence interval of the variables inserted in each model were calculated. 

Each model was evaluated by deviance analysis, predictive index, specificity and sensitivity, considering the most adequate. Thus, there were three possible physical frailty prediction models according to clinical variables for oldest-old patients of primary health care.

The study complied with national and international ethics standards for research involving human beings, following resolution no. 466/2012, approved on November 28, 2012, under registration CEP/SD: 156.413 and CAAE: 07993712.8.0000.0102, of the Research Ethics Committee in Human Beings of the Sector of Health Sciences of the Federal University of Paraná. 

## Results

The final sample consisted of 243 oldest-old individuals, with a predominance of females (161, 66.3%), and minimum and maximum age of 80 and 98 years (mean=84.4±3.8). There was a predominance of widowed (158; 65%), with low level of education (137; 56.4%) and who lived with relatives (144; 59.3%). 

Of the total sample, 36 (14.8%) were classified as Frail, 52 (21.4%) as Non-Frail and 155 (63.8%) as Pre-Frail. The majority of patients reported a disease (236, 97.1%), did not report previous falls (132, 54.3%) or hospitalizations (193; 79.4%) and used medication (233, 95.9%). There was a significant association between physical frailty and hospitalization in the last 12 months (p=0.0454).

Regarding self-reported diseases, most reported cardiovascular disease (n=211; 86.8%) and denied musculoskeletal diseases (n=148; 60,9%), digestive diseases (n=217; 89,3%), metabolic diseases (n=165; 67.9%), respiratory diseases (n=220; 90.5%), dyslipidemia (n=188; 77.4%) and other conditions (n=191; 78,6%).

Regarding the medicines used by the participants, there was a predominance of the use of 2 or more drugs from the groups of antihypertensive, diuretic and vasodilator drugs (n=113; 46.5%). The majority did not report using medications from the other groups of drugs investigated. There was a significant association between the frailty syndrome and the group of drugs classified as antidiabetic (p=0.0248).

Table 2 presents the three logistic prediction models of physical frailty for the oldest-old, considering clinical variables.

**Table 1:** Association between physical frailty and the clinical characteristics of the participants. Curitiba, PR, Brazil, 2015

Variable	Classification	Total (%)	Non-frail (%)	Pre-frail (%)	Frail (%)	p-value*
Diseases	Yes	236(97.1)	51(98.1)	150(96.8)	35(97.2)	0.8879
	No	07(2.9)	01(1.9)	05(3.2)	01(2.8)	
Number of diseases	≤ 03	171(70.4)	35(67.3)	109(70.3)	27(75.0)	0.8671
	04 to 06	59(24.3)	15(28.9)	37(23.9)	07(19.4)	
	≥ 07	13(5.3)	02(3.8)	09(5.8)	02(5.6)	
Falls in the last 12 months	Yes	111(45.7)	17(32.7)	75(48.4)	19(52.8)	0.0942
	No	132(54.3)	35(67.3)	80(51.6)	17(47.2)	
Use of medication	Yes	233(95.9)	50(96.2)	149(96.1)	34(94.4)	0.8948
	No	10(4.1)	02(3.8)	06(3.9)	02(5.6)	
Number of medicines	≤ 04	153(63.0)	35(67.3)	93(60.0)	25(69.4)	0.4376
	≥ 05	90(37.0)	17(32.7)	62(40.0)	11(30.6)	
Hospitalization in the last 12 months	Yes	50(20.6)	06(11.5)	32(20.6)	12(33.3)	0.0454
	No	193(79.4)	46(88.5)	123(79.4)	24(66.7)	
Total		243(100)	52(100)	155(100)	36(100)	

*Chi-square test, p<0.05

**Table 2:** Physical frailty prediction model for the oldest-old, according to clinical variables. Curitiba, PR, Brasil, 2015

Variables	Complete Model OR*(95%CI)	*p-value* ^†^	Model 1 OR*(95%CI)	*p-value* ^†^	Model 2 OR*(95%CI)	*p-value* ^†^
	*p*=0.303		*p*=0.013		*p*=0.115	
Metabolic diseases	2.34 (1.03-5.28)	0.041	1.99 (0.94-4.24)	0,073	2.24 (1.02-4.97)	0.045
Dyslipidemia	0.31 (0.10-1.01)	0.052	0.32 (0.11-0.99)	0,048	0.33 (0.11-1.04)	0.058
Hospitalization in the last 12 months	2.62 (1.09-6.28)	0.031	2.50 (1.13-5.57)	0,024	2.59 (1.11-6.08)	0.028
Cardiovascular Diseases	0.72 (0.24-2.18)	0.557			0.70 (0.24-2.11)	0.531
Muscoskeletal Diseases	0.81 (0.35-1.86)	0.615			0.82 (0.37-1.87)	0.651
Falls in the last 12 months	1.35 (0.62-2.92)	0.451			1.38 (0.65-2.95)	0.397
Other diseases	0.57 (0.21-1.55)	0.269			0.59 (0.22-1.59)	0.295
Number of medicines^‡^	1.44 (0.59-3.50)	0.422			1.44 (0.61-3.39)	0.399
Hearing Diseases	1.85 (0.60-5.76)	0.286			1.83 (0.61-5.54)	0.284
Use of medications	1.16 (0.18-7.40)	0.879			1.17 (0.19-7.29)	0.869
Respiratory diseases	0.93 (0.23-3.79)	0.921				
Vision diseases	1.41 (0.55-3.58)	0.472				
Urological Diseases	1.17 (0.29-4.76)	0.823				
Gastrointestinal tract diseases	0.77 (0.19-3.12)	0.717				

*OR - *odds ratio;* † Chi-square test, p <0.05; ‡ The classification of 5 or more drugs was considered

The Complete Model had a worse performance in comparison to the others, as it did not show statistical significance (p=0.303) and obtained low rates of adjustment of Frail (20.6%) and Non-frail (88.7%) and high rates of false frail (35.2%) and non-frail (47.2%). Models 1 and 2 are similar in predictive capacity (65% - 65.8%), sensitivity (55.5% - 58.3%) and specificity (66.6% - 67.1%) (Table 3). 

**Table 3:** Comparison of physical frailty prediction models for the oldest-old, according to clinical variables. Curitiba, PR, Brasil, 2015

Measures	Complete model	Model 1	Model 2
*p*-value	0.303	0.013	0.115
Prediction model	0.629	0.650	0.658
Frail - positive	0.206	0.224	0.235
Non-frail - positive	0.887	0.896	0.902
False frail	0.352	0.333	0.328
False non-frail	0.472	0.444	0.416
Sensitivity	0.527	0.555	0.583
Specificity	0.647	0.666	0.671

Model 1 stands out from the others because it presents statistical significance (p=0.013) associated with a smaller number of clinical variables in comparison with the other models (Table 2). Therefore, it was the most effective for predicting frailty in older adults in the present study.

In this model, there was statistical association only for “dyslipidemias” (p=0.048) and “hospitalization in the last 12 months” (p=0.024) (Table 2). Evaluating the OR of the variables in this model and keeping the others constant, the effect of the variable “hospitalization in the last 12 months” on variations in the prevalence of frailty can be highlighted, while the variable “dyslipidemia” (OR=0.32) has lower influence and the variable “metabolic diseases” (p=0.073; CI 0.94-4.24) has no influence in the chosen model.

## Discussion

The prevalence of frailty among the oldest-old found in this study is slightly different from the results obtained in an international systematic review, which investigated the same index among older adults aged 60 and over who lived in communities in Latin American and Caribbean countries (19.6% frail)^20^. Another international review that assessed the prevalence of the syndrome in developing countries found a variation of 17% to 31% in Brazilian studies with similar samples^21^. When considering the distribution of physical frailty by age group, especially in the group of the oldest old, the results of the present study are similar to those obtained in a cross-sectional study of the Frailty Network of Brazilian Elderly (FIBRA), carried out in seven cities in Brazil, which revealed that among 512 oldest old, 19.7% were frail and 57.2% were pre-frail^22^.

The variability of the prevalence of the syndrome may be related to the geographic locations of the samples from the studies evaluated. Likewise, the characteristics of the individuals evaluated in the present study, who are users of Basic Health Units, may be determinant for the prevention of frailty and for stability or its cure. A meticulous care provided by the health team to this age group, through pharmacological and non-pharmacological therapy, can lead to adequate management of chronic diseases, minimizing the development of possible complications from comorbidities, such as physical frailty.

In the present study, the group of drugs that was significantly associated with the development of the syndrome was the antidiabetics. The mechanisms of the association between diabetes mellitus (DM) and frailty are still uncertain^23^; however, there is evidence that DM is a potential risk factor for the development of the syndrome.

An international prospective study with 1750 older adults in Spain found an increased risk (OR 2.18, 95% CI, 1.42-3.37) of frailty in participants with diabetes. In addition, it pointed out that the use of antidiabetic medication reduced the risk to 1.01 (95% CI, 0.46-2.20)^23^. The use of medications of this class by the oldest old may contribute to the maintenance of lean mass, muscular strength and functional capacity^24^. Therefore, the control of glycemic indexes is a fundamental goal in the management of physical frailty in the oldest old.

In the final regression model, the participants who were more likely to become frail had had a hospitalization in the last 12 months (OR=2.50), dyslipidemia (OR=0.32) and metabolic disease (OR=1.99).

The association of the syndrome with hospitalization in the last 12 months was highlighted in national^10^ and international^8^ ^,^ ^25^ ^-^ ^26^ ^)^ authors. A systematic review evaluated 31 international articles and found that frailty increases the risk of hospitalization from 1.2 to 1.8 times^25^. This finding is similar to another cross-sectional study carried out with 993 older adults aged 70 years or older residing in Albacete, Spain, which found a 1.7 times increased risk of hospitalization^26^. Physical frailty generates a greater demand for care due to the reduced capacity of response to several stressors and the decrease in the of homeostasis, which causes negative health outcomes, such as hospitalization. 

The high chances of hospitalization in the present study are possibly related to the age range of the sample. There is a scarcity of national and international studies that exclusively address the oldest old. This approach is necessary due to the peculiarities of this age group, which are different from those of younger adults, especially due to higher rates of negative health outcomes. 

Regarding the variable “dyslipidemia”, which was associated with greater probability of physical frailty in this study, international authors^23^ ^,^ ^27^ ^-^ ^28^ highlighted the relationship between this factor, sarcopenia and other morbidities, especially Diabetes Mellitus and cardiovascular diseases. Dyslipidemia associated with other chronic diseases favors the occurrence of neuromuscular changes and, consequently, leads to changes in walking speed, balance and to the physical frailty syndrome^28^ ^-^ ^29^. 

Regarding the influence of the variable “metabolic disease” in the predictive model, it is possibly related to neuroendocrine dysregulation, one of the factors that leads to the development of physical frailty^30^. Hormonal alterations^31^ and hypovitaminosis^32^ have been identified as important disorders associated with the syndrome.

Vitamin D can be highlighted for its role in the musculoskeletal health of older adults and its consequent relationship with the sarcopenic process. A prospective international study with 727 older adults aged 65 years and over in the Augsburg region of Germany found that participants with low vitamin D levels had significantly higher odds of developing the syndrome (OR=2.53) when compared to those with normal levels^32^. In this sense, orientation and encouragement regarding exposure to the sun, intake of food rich in vitamin D and practice of physical exercises is considered a nursing role. 

For gerontological nursing, the elaboration of a physical frailty prediction model contributes to a greater objectivity in the screening of the oldest old^33^. This is the fastest growing age group in the world; they have characteristics different from younger older adults and are often excluded from scientific studies. Investigations addressing subjects aged 80 and over should be stimulated in order to increase knowledge about the prevalence of syndromes, associated factors, and health and disease conditions in this age group. 

The results of this study include clinical factors that may interfere in the development of the syndrome and represent possible intervention factors in gerontological nursing care. In this context, the elaboration of a prediction model is the first step for planning care to minimize the development of frailty and establishing interventions to maintain functional capacity and adequately manage the syndrome. The evaluation of the odds of an older adult becoming frail can support a decision-making process based on clinical reasoning aimed at the prevention of the health problems of the oldest old, even in primary care.

Regarding the limitations of this research, its cross-sectional design means it is not possible to establish causal relations between the clinical variables and the outcome of this investigation. In addition, the sample represents a specific community, so the results cannot be generalized. Longitudinal and multi-center studies should be conducted to deepen the investigation of these relationships and to verify the transitions between levels of frailty in relation to severity and reversibility of cases in the medium and long term.

## Conclusion

The present study proposed a Physical Frailty Prediction Model for the oldest old according to clinical variables, which included “metabolic disease”, “dyslipidemia” and “hospitalization in the last 12 months”. In the univariate analysis of the data, the clinical variables “hospitalization in the last 12 months” and “antidiabetics” were associated with the development of the physical frailty syndrome.

Regarding the management of physical frailty in primary care, the nurse must provide an assistance that addresses the peculiarities of the oldest old and develop actions aimed at the prevention of the syndrome and related clinical factors. Nursing interventions in primary care, such as encouraging physical activity, providing orientation on adequate nutritional intake and clarification about the correct use of medications and conducting clinical follow-up of the elderly are important strategies for the maintenance of lean mass, muscular strength, functional capacity, and lipid levels, which in turn favor the reduction of important clinical factors, such as dyslipidemia and hospitalizations. In addition, these measures allow the monitoring of non-frail and pre-frail elderly individuals in order to reduce transition to more severe levels of the syndrome.

For the present study, the choice of a physical frailty prediction model for the oldest old provides a faster, less expensive clinical application, without the need for a differentiated environment for the evaluation of certain markers. In addition, it reduces the use of specific equipment to screen for the syndrome. The choice of a physical frailty regression model is the first step in the elaboration of clinical nursing methods to evaluate the oldest old in primary care.
